# Expression of Rice CYP450-Like Gene (*Os08g01480*) in *Arabidopsis* Modulates Regulatory Network Leading to Heavy Metal and Other Abiotic Stress Tolerance

**DOI:** 10.1371/journal.pone.0138574

**Published:** 2015-09-24

**Authors:** Arti Rai, Ruchi Singh, Pramod Arvind Shirke, Rudra Deo Tripathi, Prabodh Kumar Trivedi, Debasis Chakrabarty

**Affiliations:** 1 Council of Scientific and Industrial Research—National Botanical Research Institute (CSIR-NBRI), Rana Pratap Marg, Lucknow, 226001, India; 2 Academy of Scientific and Innovative Research (AcSIR), Anusandhan Bhawan, 2 Rafi Marg, New Delhi, 110 001, India; National Institute of Plant Genome Research, INDIA

## Abstract

Heavy metal (HM) toxicity has become a grave problem in the world since it leads to hazardous effects on living organisms. Transcriptomic/proteomic studies in plants have identified a large number of metal-responsive gene families. Of these, cytochrome-P450 (CYPs) family members are composed of enzymes carrying out detoxification of exogenous molecules. Here, we report a CYP-like protein encoded by *Os08g01480* locus in rice that helps the plant to combat HM and other abiotic stresses. To functionally characterize CYP-like gene, cDNA and promoter were isolated from rice to develop *Arabidopsis* transgenic lines. Heterologous expression of *Os08g01480* in *Arabidopsis* provided significant tolerance towards abiotic stresses. *In silico* analysis reveals that *Os08g01480* might help plants to combat environmental stress via modulating auxin metabolism. Transgenic lines expressing reporter gene under control of *Os08g01480* promoter demonstrated differential promoter activity in different tissues during environmental stresses. These studies indicated that differential expression of *Os08g01480* might be modulating response of plants towards environmental stresses as well as in different developmental stages.

## Introduction

Plants are sessile organisms, exposed to adverse environmental conditions throughout their life, which in turns influences negative effects on growth and development process and/or productivity. These environmental stress conditions are caused from various anthropogenic activities including mining industries, foundries, smelters, coal-burning power plants and many agricultural practices. These activities cause accumulation of toxic compounds in the rhizosphere, salinity and degradation of the soil, and environmental pollution and climate change [1,2]. Among various toxic compounds heavy metals (HMs) are important factors which are present in agricultural land and lead to physiological and metabolic alterations in plants [3].

HM are categorized as essential and non essential elements for the organisms under physiological circumstances [4]. The main threats to human health from HMs are associated with exposure to cadmium (Cd), lead (Pb), chromium (Cr), mercury (Hg) and arsenic (As). Though HMs is present in most foodstuffs, their exposure to human beings varies considerably due to differences in dietary habits. Similar to other stresses, HM exposure also induces oxidative stress and modulates gene expression [5–7].

HMs are highly reactive and thus interact with thiols or other groups to disturb many physiological processes by interfering with enzymatic reactions [4,8]. HMs cause toxicity and modifications in plant system at molecular and cellular levels comprised of different plant physiological processes [9–10]. HM toxicity also interrupts redox homeostasis by stimulating the formation of reactive oxygen species (ROS) and free radicals like singlet oxygen (^1^O_2_), superoxide radicals (O_2_^•-^), hydrogen peroxide (H_2_O_2_) and hydroxyl radicals (^•^OH) [11–12]. HMs tolerance mechanisms in plant necessitate synchronization of physiological and biochemical pathways, and changes in transcriptome profile at stress point [6,13]. To cope with hazardous effects of HMs, plants employ various strategies. Tolerance to deleterious effect involves chelation of HMs with different ligands, metallothioneins, phytochelatins, compartmentalization in vacuoles and start up of actively up-regulated antioxidant machinery [14–16].

In past, different studies involving sensitive, tolerant, mutant and hyper-accumulator plants in the genre of physiology, genomics, proteomics and metabolomics suggested different strategies for HM tolerance [8]. Many transcriptomic and sequencing studies led to identification of several HM induced genes in plant. These genes are cytochromes (CYPs), heat shock proteins, metallothionein, phytochelatin, various transporters, regulatory genes, structural genes etc. However, in order to gain insight of these induced proteins for their function, it is necessary to characterize them functionally. In recent past, some of genes identified through transcriptome studies have been functionally characterized to decipher their involvement in HM stress [17–20]. In our previous study, we reported that *Os08g01480* was up-regulated in HM stress and specifically in As stress in rice [6]. Further, it was also shown that *Os08g01480* up-regulation was more in As tolerant than sensitive genotypes of rice [21–22]. Together, these observations lead to the hypothesis that, in rice, *Os08g01480* might play an important role in As stress metabolism. The predicted *Os08g01480* protein appears to be a cytochrome P450 monooxygenase that however does not contain EXXR motifs present in other CYPs. Few studies suggest that this motif from CYP is not as essential to CYP architecture as previously thought [23]. The present study elucidates the molecular function of As-responsive CYP like gene (*Os08g01480)* of rice in plant growth, development and under different stress conditions through developing transgenic *Arabidopsis* lines.

## Materials and Methods

### Plant materials and growth conditions

*Arabidopsis thaliana* (L.) accession Col-0 plants were grown in growth chamber (Canviron, USA) under controlled conditions 16h light-8h dark photoperiod cycle, 22°C temperature, 150–180 μmE m^-2^ s^-1^ light intensity and 60% relative humidity. Seeds were sown in soilrite and stratified for 48 h in the dark at 4°C before shifting in the light. Plants were watered with nutrient media and water at alternate interval in every week until plants complete their lifecycle.

### Expression profiling and phylogenetic analysis

Publicly available CEL files in the GEO database was used for digital expression analysis with the help of dCHIP software [24]. Analysis of *Os08g01480* during As(V) stress in rice *japonica* varieties Azucena and Bala has been carried out using the CEL files (GSE4471). Expression of *Os08g01480* in different developmental stages has been analyzed using CEL files (GSE6893, GSE7951 and GSE14304). GEO databases for expression data related to stress treatment (GSE6901) was used for expression analysis of the rice *Os08g01480* gene (Table A in S1 File). Amino acid sequences of all *Arabidopsis* CYPs were retrieved from TAIR and rice *Os08g01480* sequence was retrieved from Rice Genome Annotation Project (http://rice.plantbiology.msu.edu/). Alignment of amino acid sequence has been performed by ClustalW. Phylogenetic relationships were inferred using the maximum likelihood (ML) method. For phylogenetic analysis of all identified CYPs, the JTT substitution model was used and results were evaluated with MEGA v5.2 with 1000 bootstrap replicates Cloning, construct preparation and transgenic plant development.

Full-length cDNA sequence of *Os08g01480* (1173 bp) was obtained from Rice Genome Annotation Project. *Os08g01480*, construct was prepared using cDNA library prepared from rice root and nested primers (Table B in S1 File). Simultaneously, *Os08g01480* was cloned in plant expression vector pBI121 containing constitutive promoter CaMV35S between *Xba*1 and *Sac*1 using specific primers (Table B in S1 File). This construct was transformed in *Agrobacterium tumefaciens* strain GV3101 by the freeze thaw method [25]. *Arabidopsis* transgenic lines were generated through floral dip method [26]. Transformants were screened on ½ MS agar plates containing kanamycin (50 μg ml^-1^). Based on the relative expression of transgene through semi-quantitative RT-PCR and qRT-PCR, three lines were selected for analysis. All the studies were performed in T4 homozygous lines of *Arabidopsis*. Promoter of *Os08g01480* (500 bp) was cloned in pTZ57R/T cloning vector using rice genomic DNA using nested primers (Table B in S1 File). It was cloned in pBI121 plant expression vector between restriction sites *Hin*dIII and *Bam*HI using specific primers (Table B in S1 File). This construct was used for generation of transgenic lines of *Os08g01480* promoter.

### Analysis of transgenic plants

Phenotypic variations in transgenic lines of *Arabidopsis* expressing *Os08g01480* were monitored during complete life cycle and different phenotypic characters were collected at indicated growth stages and in at least 2–3 successive generations. For histochemical GUS staining activity, promoter *Os08g01480*:*uidA* seedlings and different tissues were incubated 24 h at 37°C in GUS staining buffer containing 100 mM Na_3_PO_4_, 1.0 mM ferrocyanide, 1.0 mM ferricyanide, 0.05% Triton X-100, pH 7.2, and 2 mM 5-bromo-4-chloro-3-indolyl-b-D-glucuronide (initially dissolved in N,N-Dimethyl formamide) [27]. After successive washing from the gradient of ethanol (70%- 90%), images were captured under light microscope (Carl Zeis, USA).

### Quantification of metal accumulation

WT and transgenic lines were grown in the ½ MS plates supplemented with different concentration of HMs. Whole seedling of WT and transgenic lines were separated manually and washed in MQ water at-least four times to remove surface and apoplastic HMs. All samples were dried out in hot air oven at 40°C till it weighed constant. Dried plants tissues (100 mg) were digested in HNO_3_ and H_2_O_2_, 3:1 ratio employing microwave digestion [28], in CEM-MDS 2000 Microwave digester (SpectraLab Scientific Inc., Canada). HMs were quantified using atomic absorption spectrometry (AAS) (Perkin-Elmer; AAnalyst 600) as per standard protocol [7,18].

### Chlorophyll fluorescence measurements

An Imaging-PAM, M-Series Chlorophyll Fluorometer (Walz, Effeltrich, Germany) was used to study the chlorophyll fluorescence parameters in transgenic lines and WT. Calculations of various chlorophyll fluorescence parameters were done according to standard protocol of Maxwell [29]. The maximum photochemical efficiency of photosystem II (PSII), [Fv/Fm], effective quantum yield of PSII [Y(II)], non photochemical quenching (NPQ) (efficiency of excess excitation energy dissipation by heat). NPQ (photo inhibition), Y (NPQ) [photons absorbed by PSII antennae] and Y(NO) (irreversible PSII damage) parameters was studied [29].

### Pathway analysis

Pathway Studio version 7.0 (Ariadne Genomics, USA) was used for the construction of cellular pathway and transcriptome interactions for *Os08g01480*. The pathway diagram was further filtered to show only proteins/transcription factors/metabolites involved in cellular processes associated with development and stresses.

### Expression analysis by qRT-PCR

About 5 μg of DNA free total RNA isolated from different rice and *Arabidopsis* samples was used to synthesize first strand cDNA using SuperScript II (Fermentas, USA), following the manufacturer’s recommendations. This synthesized cDNA used to accomplish qRT-PCR for few of selected genes using SYBR Green Supermix (ABI Biosystems, USA) in an ABI 7500 instrument (ABI Biosystems, USA). *Actin* of rice and *Arabidopsis* was used as an internal control for setting up equal amount of cDNA in all the reactions.

### Statistical analysis

Each experiment was done according to completely randomized design with three independent experiments with at least three replications. The data proceeded by two way analysis of variance (ANOVA) to authenticate the variability and validity of results, and Duncan’s Multiple Range Test (DMRT) was achieved to detect the significant difference between treatments [30].

## Results

### *In silico* analysis

The annotated ORF of *Os08g01480* in TIGR database is 1173 nucleotides which encodes 390 amino acids protein. The studied gene was found to be part of CYP450 super family (cl12078) and it consist the encoded protein of CYP450 domain with interval from 67–390 amino acids. The evolutionary relation of *0s08g01480* was assessed through phylogenetic study using *Arabidopsis thaliana* A-type and non-A type CYP sequences. A maximum likelihood phylogenetic tree was generated using MEGA v5.2 based on the alignment of polypeptides encoded by all identified CYPs of *Arabidopsis* and *0s08g01480* (Fig A in S1 File). CYP71B formed largest CYP cluster in phylogenetic analysis. *Os08g01480* clustered with A type *Arabidopsis*, CYP71A subfamily and is closely grouped with CYP71A22, CYP71A2, CYP71A23 and CYP71A26 under CYP71A subfamily in this analysis.

### Stress-responsive expression of *Os08g01480*

Expression profiling analysis of *Os08g01480* in Azucena [As(V)-sensitive] and Bala [As(V)-tolerant] rice varieties using publicly available GEO database showed that this gene is up-regulated in As tolerant variety, Bala in comparison to sensitive variety Azucena [13]. In a study of four rice cultivars during As(III) and As(V) stress higher level of gene expression of *Os08g01480* was found in Triguna, IR-36 and PNR-519 (As-tolerant) compared to IET-4786 (As-sensitive) [21]. Another study using six contrasting rice genotypes (BRG-12, BRG-15, BRG-20, CN1646-5, Nayanmoni and CN1646-2) showed that *Os08g01480* was up-regulated in high As accumulating rice genotypes (BRG-12 and BRG-15) and down-regulated in low As accumulating rice genotypes (Nayanmoni and CN1646-2) [22]. The microarray dataset available for drought, salt, cold stresses also showed involvement of *Os08g01480* gene during these abiotic stresses in rice [31] (Fig B in S1 File).

### Altered growth of the transgenic *Arabidopsis* lines

Three independent homozygous transgenic lines with significantly higher *Os08g01480* expression were selected for further characterization. In early stages of vegetative growth on Soilrite, no phenotypic difference was observed in transgenic lines in comparison to WT. However, there was clear difference in bolting time between WT and transgenic lines (Fig C in S1 File). The visible changes in early plant growth between WT and transgenic lines were analyzed through measuring root length after growth on ½ MS plates for 11 days. All the transgenic lines showed significantly increased root length compared to WT plants. Moreover; early germination rate of WT and transgenic lines was also compared. Observation suggests enhanced germination rate in transgenic lines as compared to WT. There was ~30% increase of seed germination in transgenic lines after 1 d and 2 d. While on 3 d, transgenic lines showed ~20% of increase in germination in comparison to WT (Fig D in S1 File).

### Abiotic stress response of the transgenic *Arabidopsis* lines

**Fig 1:**
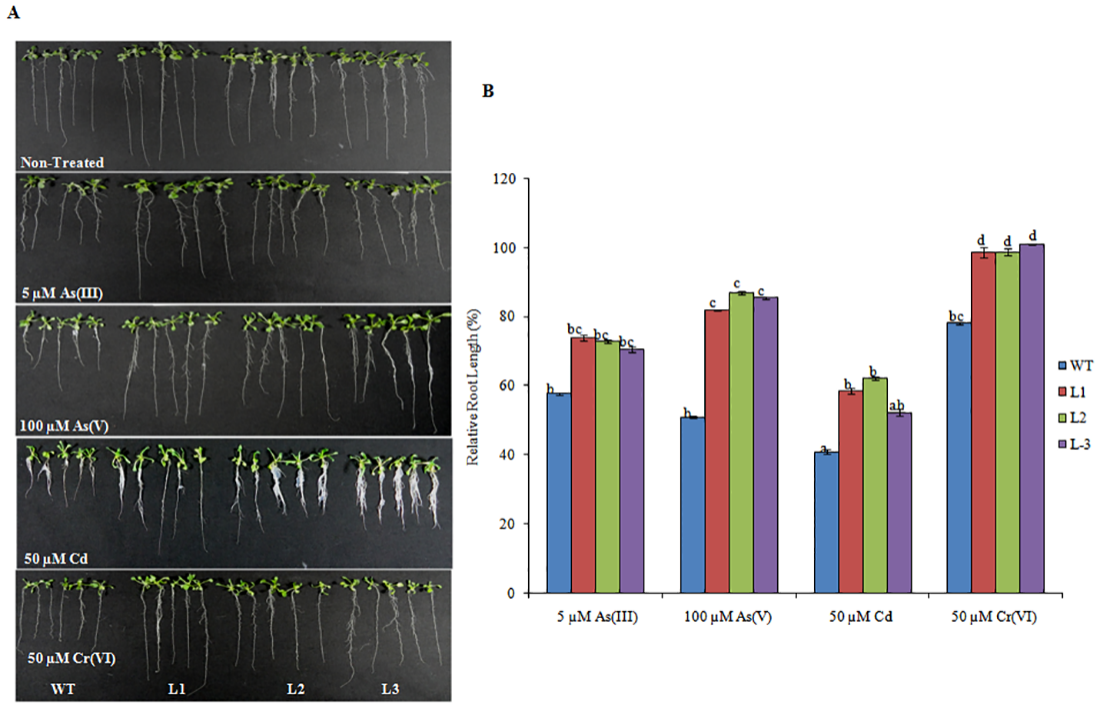
Expression of *Os08g01480* in *Arabidopsis* confers tolerance for HMs. Seeds of WT (Col-0) and transgenic lines (L1, L2 and L3) were germinated vertically for 11 d on ½ MS medium supplemented with 5 μM As(III), 100 μM As(V), 50 μM Cd and 50 μM Cr(VI). (A) Phenotypic changes of WT and transgenic lines grown in different HMs. (B) Relative Root length of the WT and transgenic plants during stress conditions in comparison to WT and transgenic plants grown in non treated media. All values are the mean of triplicates (±SD) Values marked with similar letters are not significantly (Duncan’s test: p<0.05) different.

To determine response of *Os08g01480* in stress, transgenic *Arabidopsis* lines were germinated on ½ MS media containing different concentrations of HMs for 11 d. Significant root growth inhibition was observed in WT and transgenic lines exposed to different HM concentration. However, tolerance was observed in transgenic lines as compared to WT plants (Fig 1). During 5 μM As(III), 100 μM As(V), 50 μM Cd, and 50 μM Cr(VI) stress, there was ~40%, 50%, 55%, and 20% relative root length as compared to non-treated (NT) decrease in WT respectively. At the same time, significantly lower (~25%, 20%, 40%, and 7%, decrease) root length inhibition in transgenic plants was recorded in comparison to root length of NT transgenic plants (Fig 1).

**Fig 2:**
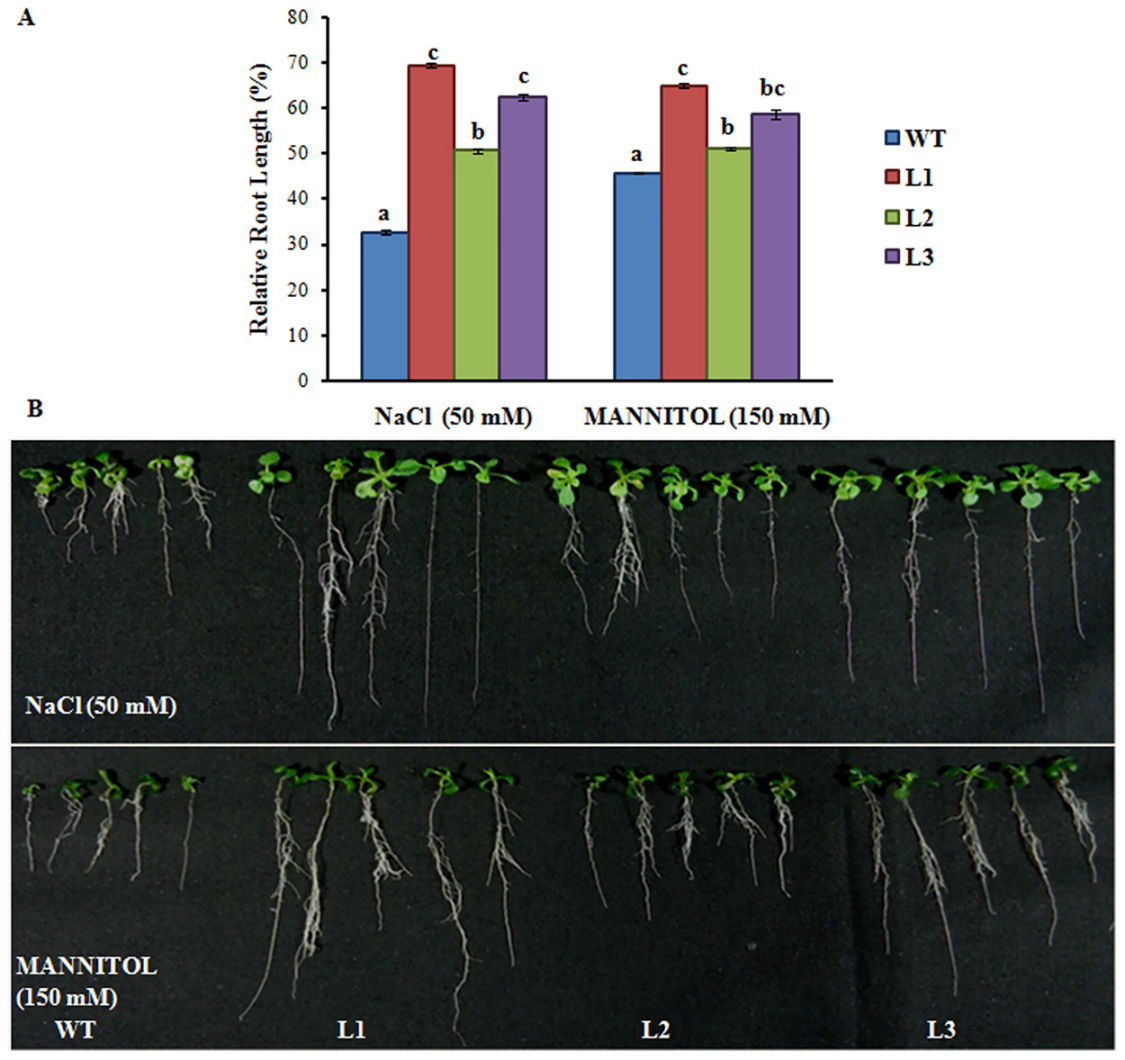
Expression of *Os08g01480* in *Arabidopsis* confers tolerance for salt and osmotic stress. (A) Relative root length of the WT (Col-0) and transgenic plants after vertical growth of 11 d on ½ MS media plates supplemented with NaCl and Mannitol in comparison to WT and transgenic lines grown in non treated media. All values are the mean of triplicates (±SD) Values marked with similar letters are not significantly (Duncan’s test: p<0.05) different. (B) Phenotypic changes of WT and transgenic lines grown in ½ MS medium supplemented with NaCl and Mannitol.

For salt and osmotic stress, WT and transgenic line were grown in NaCl (50 mM) and mannitol (150 mM) for 11 d. Root length of WT showed significant decrease (70%) as compared to transgenic line (30%-50%) during salt stress as compared to NT; WT and transgenic seedlings, whereas in osmotic stress, ~50% and up to 40% decrease was observed in WT and transgenic lines compared to NT; WT and transgenic plants. It is concluded that in WT there was ~40% and 20% more reduction in case of salt stress and osmotic stress respectively (Fig 2).

**Fig 3:**
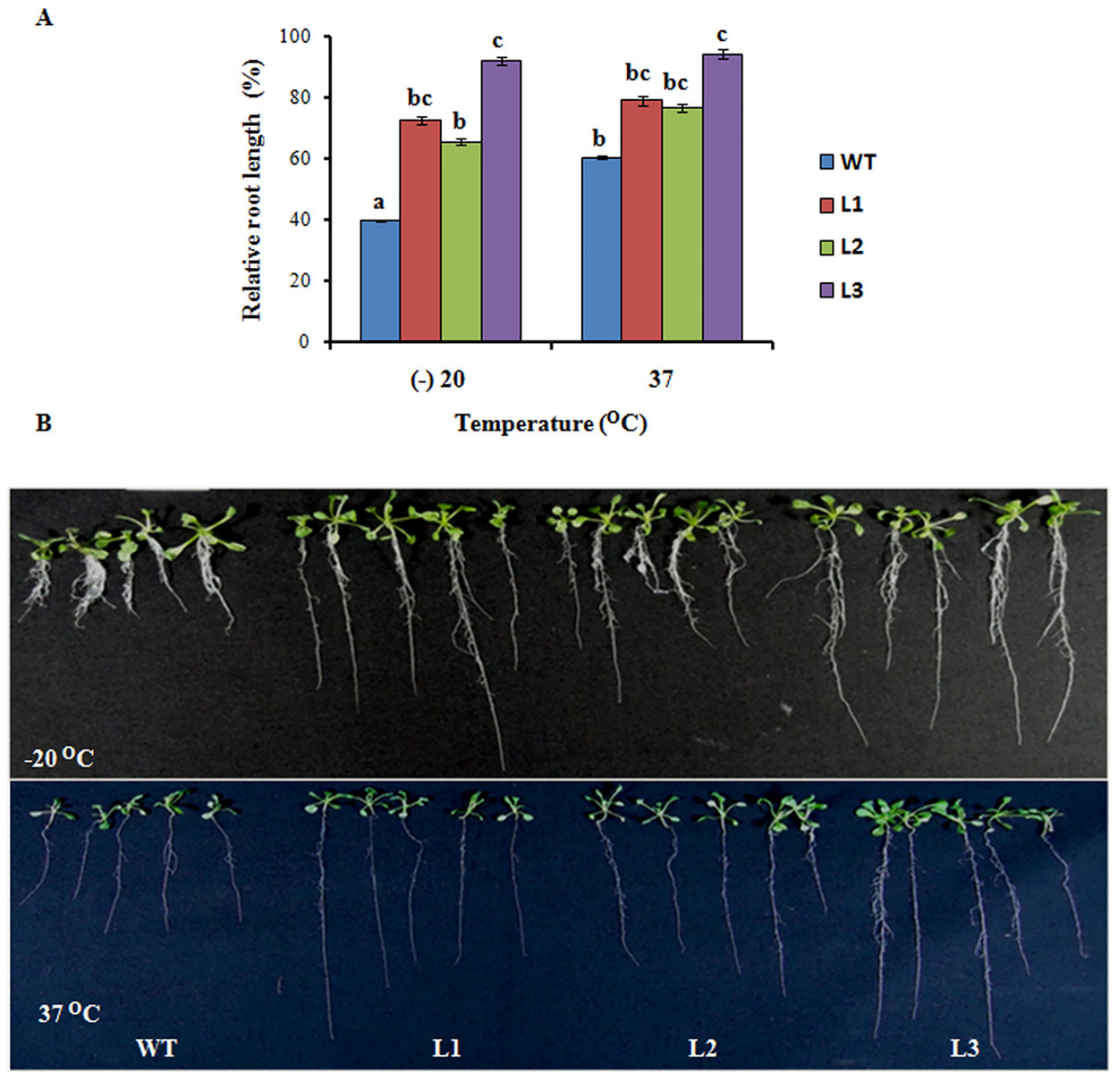
Expression of *Os08g01480* in *Arabidopsis* confers tolerance to temperature stress. (A) Relative root length of the WT (Col-0) and transgenic plants after vertical growth of 11 d on ½ MS media plates after exposure at -20°C and 37°C for 4h in comparison to WT and transgenic lines grown in non treated media. (B) Phenotypic changes of WT and transgenic lines grown after exposure at -20°C and 37°C All values are the mean of triplicates (±SD) Values marked with similar letters are not significantly (Duncan’s test: p<0.05) different.

For studying effect of cold and heat, after stratification seeds were kept at -20°C and 37°C respectively for 4 h and then transferred to control conditions. There was ~60% and 40% decrease in root length of WT during cold and heat stress, respectively in comparison to non treated WT. On the other hand only ~25% and ~15% decrease of root length was recorded in transgenic lines at time of heat and cold stress, respectively when compared to NT transgenic plants. This result indicated ~40% and ~20% reduction in root length of WT compared to transgenic lines subjected to cold and heat stresses (Fig 3). We also observed increased lateral roots in transgenic lines especially in Line 1 and 3 (as expression level of *Os08g01480* is higher in these lines) under different abiotic stress (Figs 1–3). These results indicate that *Os08g01480* promotes lateral root development in transgenic lines which is an adoptive mechanism to various stress conditions. All these studies suggest that *Arabidopsis* expressing *Os08g01480* exhibit tolerance against different environmental stresses.

### Metal accumulation and transporters expression analysis

To analyze whether the tolerance observed in the transgenic lines is due to modulated metal accumulation potential, WT and transgenic *Arabidopsis* lines were grown in media supplemented with different concentrations of metals for two weeks. This analysis demonstrates that there was differential accumulation pattern of HMs in WT and transgenic plants. No significant change in metal accumulation was noticed in WT and transgenic seedlings grown in media supplemented with As(III), Cd and Cr(VI). Moreover; increase in total As accumulation was observed in transgenic lines when exposed to As(V). Further, to establish increase in As accumulation was due to increased expression of phosphate transporters (*AtPHT1*) and/or ABCC transporters (*AtABCC1* and *AtABCC2*), qRT-PCR analysis was performed. Surprisingly, *AtPHT1*, *AtABCC1* and *AtABCC2* expression was up-regulated in transgenic plants (Fig E in S1 File).

### Chlorophyll fluorescence analysis

The chlorophyll fluorescence imaging and analyses of photosystem (PSII) of WT and transgenic plants were carried out in different environmental stress conditions and represented in spider plots. Each spider plot consisted of different parameters; some measured with dark-adapted plants (Fv/Fm) while others with low light intensity (80 mmol m^-2^s^-1^). No variability of the chlorophyll fluorescence parameters in WT and transgenic lines was observed under control conditions (Fig F in S1 File). Dynamics of chlorophyll fluorescence parameters did not show much change in case of transgenic lines under stress conditions. For heavy metal stress after 14 d of growth under normal conditions in pot, WT and transgenic plants were watered with nutrient media containing 100 μM As(III), 200 μM As(V), 200 μM Cd and 400 μM Cr(VI) for two weeks and then chlorophyll fluorescence was analyzed. Spider plot of WT plants deformed during HM stresses with respect to NT plants. Transgenic plants showed increase in non-regulated energy dissipation under HM exposure which is considered as regulation of excess energy dissipation via trans-thylakoid proton gradient (ΔpH) development (Fig G in S1 File). However, WT plants showed increase in non photochemical energy dissipation [(via increased NPQ, qN, Y(NPQ)] under stress condition.

**Fig 4:**
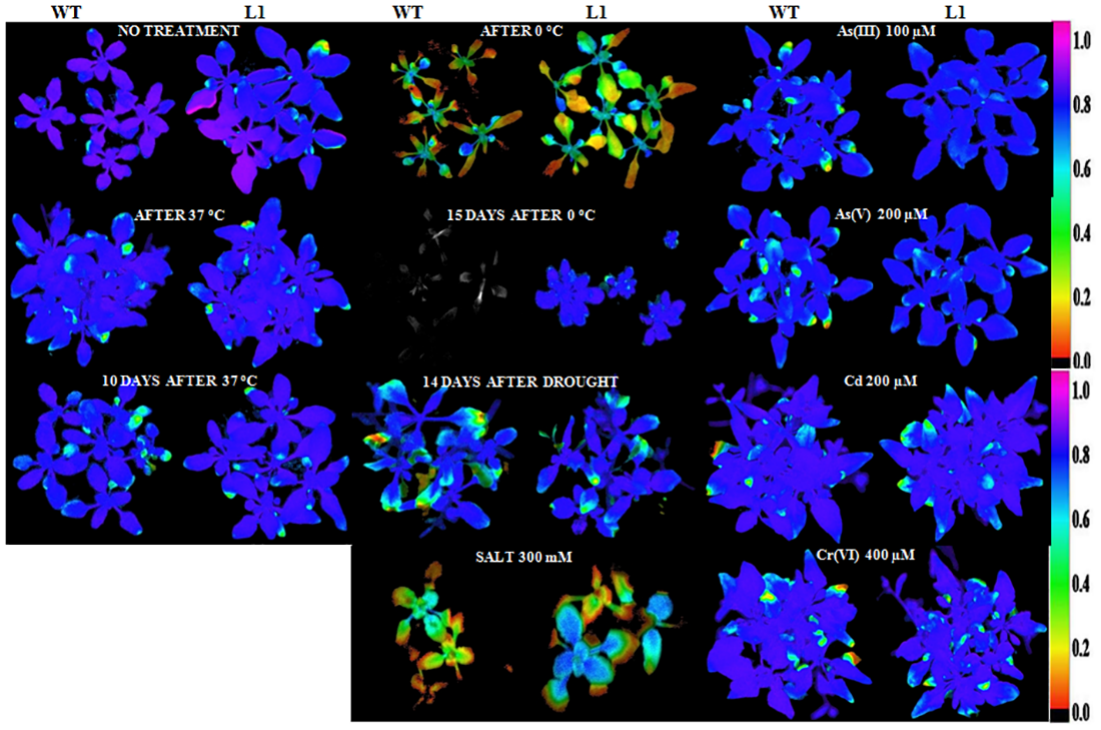
Chlorophyll fluorescence imaging (Fv/Fm) of WT and transgenic line (L1) was performed during control and stress conditions. Fv/Fm of non-treated WT and transgenic was carried out after 14 days of germination. Fv/Fm was carried out for WT and transgenic line after they were exposed to 37°C and 0°C for 4 h and again after10 and 15d recovery from heat and cold stress, respectively. For salt and different HM stress WT and transgenic line were watered with nutrient media containing NaCl (300 mM), 100 μM As(III), 200 μM As(V), 200 μM Cd and 400 μM Cr(VI) for two weeks and then Fv/Fm were measured. For osmotic stress WT and transgenic line were withheld from water for 14 d, and then rewatered for 7 d before Fv/Fm was measured. The false color code depicted on the right side of images ranges from 0 (black) to 1 (pink) Level of significance is considered as P≤0.05.

Fluorescence dynamics for cold and heat stress were performed immediately after WT and transgenic lines were kept at 0°C and 37°C for 4 h and then after 10 d and 15 d of recovery under simultaneous pot conditions. After recovery period, no significant improvement in fluorescence dynamics of WT was observed although transgenic plants recovered from deformation. Spider plot of WT plants show significant deviation in dynamics under cold, heat, osmotic and salt stress conditions while transgenic lines did not show much deviation (Fig H in S1 File). Photosynthesis efficiency is maximal photochemical quantum yield of plants is represented in pictorial form as chlorophyll fluorescence imaging during control and different stress conditions for WT and transgenic line (Fig 4). The false color code depicted on the right side of images ranges from 0 (black) to 1 (pink) Level of significance is considered as P≤0.05. These results suggested that transgenic plants are easily adaptive to stress conditions as their fluorescence dynamics did not change significantly.

### Predicted molecular network of *Os08g01480*

Pathway studio software was used to identify common molecular connections between the proteins/transcription factors/small metabolites due to expression of *Os08g01480* in *Arabidopsis*. Various cellular processes/molecular pathways were identified using pathway analysis. Pathway studio searches through the ResNet database [32] for all known interactions between genes/ proteins/ metabolites such as regulation and their expression. Each arrow indicates interactions between genes and a cell process pathway.

**Fig 5:**
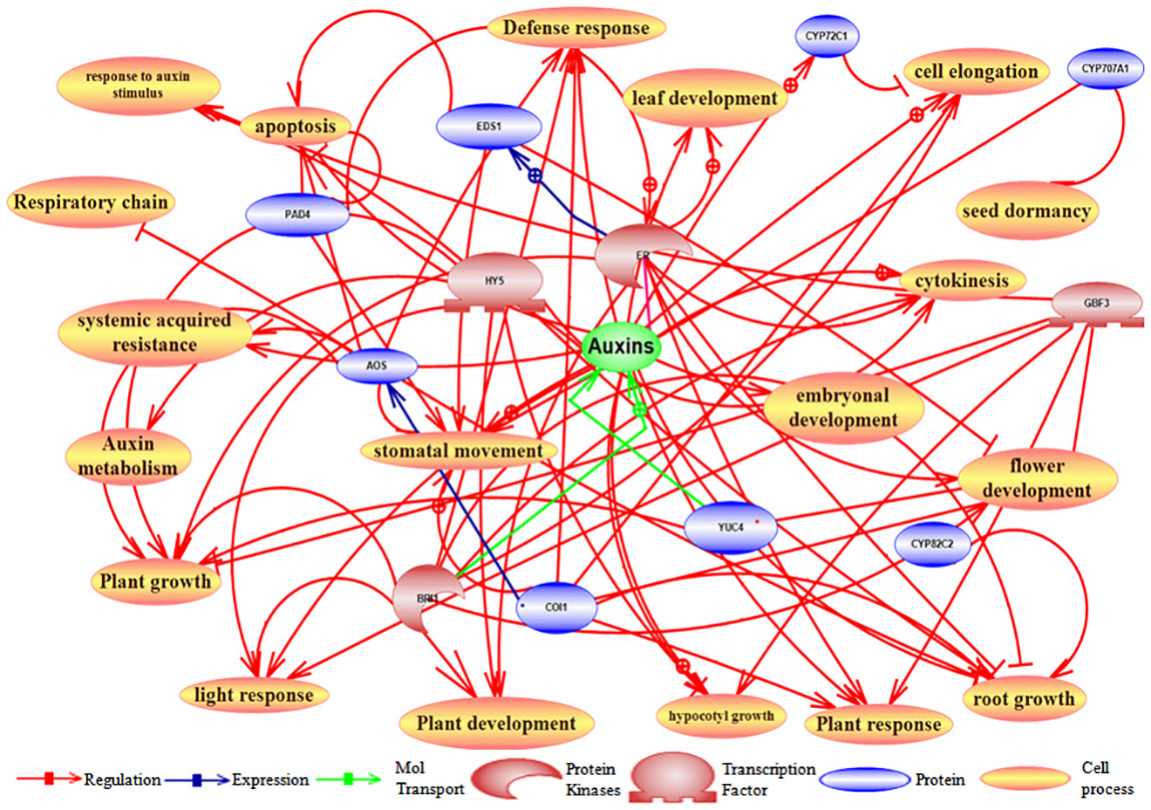
Pathway studio analysis used for prediction of molecular network modulated by expression of CYP like gene *Os08g01480* in *Arabidopsis*. Pathway studio is utilized for finding common molecular connections between the proteins/transcription factors encoded by the expressed probe sets. This software analyses through the ResNet database for all known interactions between genes/proteins such as regulation and their expression. Each arrow indicates interactions between genes and a cell process pathway.

The predicted network (Fig 5) shows that the genes involved in many cellular processes like defense response, leaf development, root growth, flower development, embyronal development, hypocotyls growth, stomatal movement, auxin metabolism, cell elongation, cytokinesis, apoptosis and light response etc. are associated by *Os08g01480*. Genes related to defense response such as *COI1*, *BRI1* and *AOS* were observed in the networks which are modulated during expression of *Os08g01480* in *Arabidopsis*. This suggests that these genes might be playing a role in improved defense response of transgenic lines against different abiotic stress. Auxin stimulus responsive genes (*HY5* and *GBF*) and gene related to systemic acquired resistance (SAR), *AOS*, was also involved in network. These genes play important role in improving plant defense mechanism and help the plant to combat abiotic stress. *EDS1*, *COI1*, *YUC4* and *ER* in flower development, *COI1* and *BRI1* in cell elongation, *CYP707A1* in stomatal movement, *EDS1* in apoptosis and *AOS*, *CYP82C2*, *COI1* and *HY5* in root growth are few genes predicted in pathway known to be involved in the growth and development of the plants.

### Expression pattern of co-regulated genes in *Os08g01480* transgenic plants

**Fig 6:**
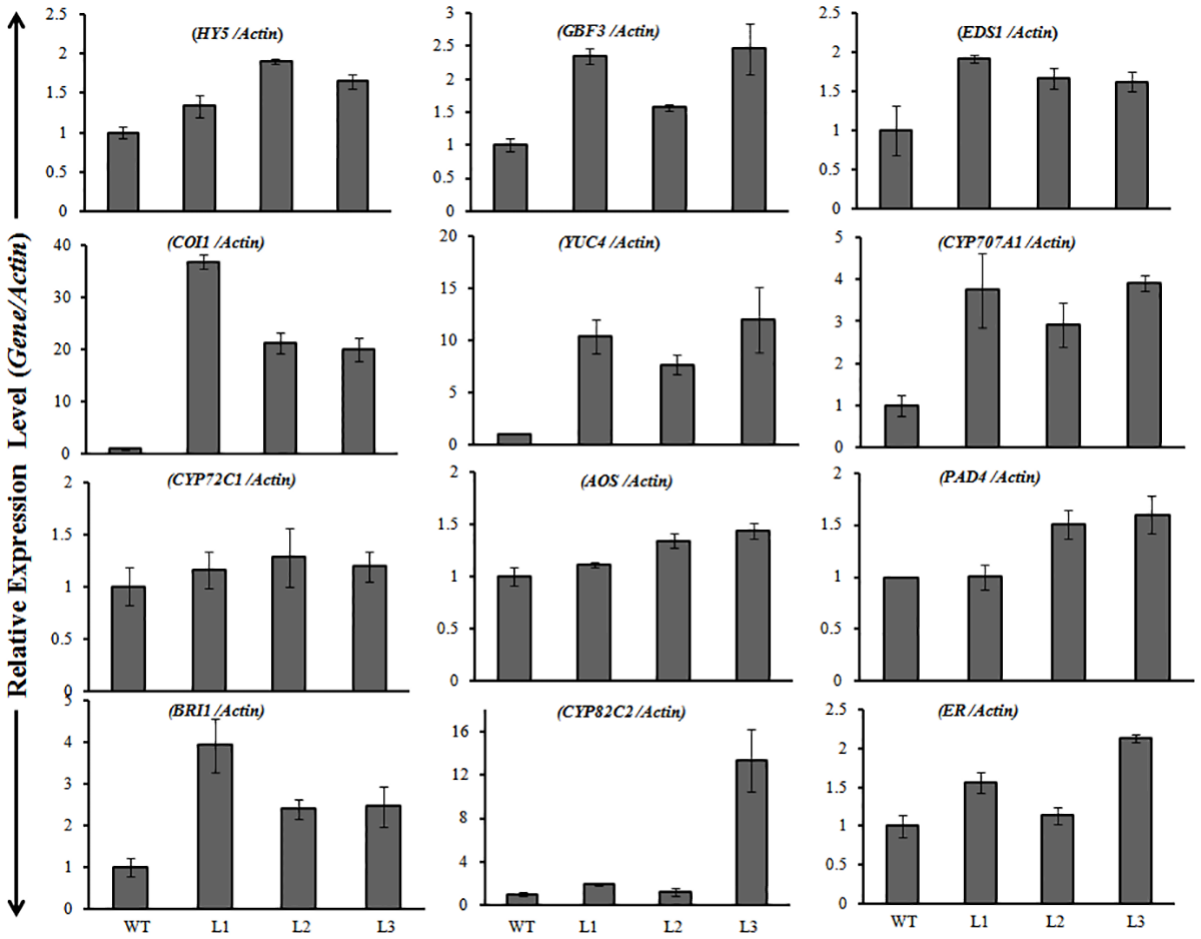
Expression analysis of co-regulated genes related to network modulated by expression of *Os08g01480* in *Arabidopsis*. qRT-PCR analysis was carried out in two week old seedlings of WT and transgenic lines grown in ½ MS media.

To elucidate the rationale behind tolerance of transgenic lines to different abiotic stresses, expression of co-regulated genes which are known to help in defense, growth and development of plants were examined. An enhanced expression of *HY5*, *GBF3*, *EDS1*, *COI1*, *YUC4*, *CYP707A1*, *CYP72C1*, *AOS*, *PAD4*, *BRI1*, *CYP82C2* and *ER* was observed in transgenic lines in comparison to WT (Fig 6). Enhanced expression of these genes seems to be possible reason for tolerance of transgenic lines against different abiotic stresses.

### Activity of *Os08g01480* promoter

*In silico* analysis of *Os08g01480* during different developmental stages of rice suggested that it is mainly expressing in seedling and panicle of rice. Interestingly, expression of the gene was more prominent during different development stages of androecium. *In silico* results were confirmed by high relative expression of *Os08g01480* in seedling and androecium (Fig I in S1 File). In addition, *in silico* analysis of *Os08g01480* promoter was performed using PLACE online tool. Presence of consensus sequences of plants *cis*-regulatory elements in promoter region of *Os08g01480* demonstrated its putative involvement during plant growth, development and defense (Table C in S1 File).

Further to validate above results, *Arabidopsis* transgenic lines expressing *uidA* gene under control of *Os08g01480* promoter were generated (Fig J in S1 File). Examination of GUS activity in different developmental stages of transgenic plants showed prominent activity in flowering tissue (androecium). Relative expression level of *uidA* was analyzed in different tissue of *Arabidopsis* through qRT-PCR and showed higher expression in flower (Fig K in S1 File).

**Fig 7:**
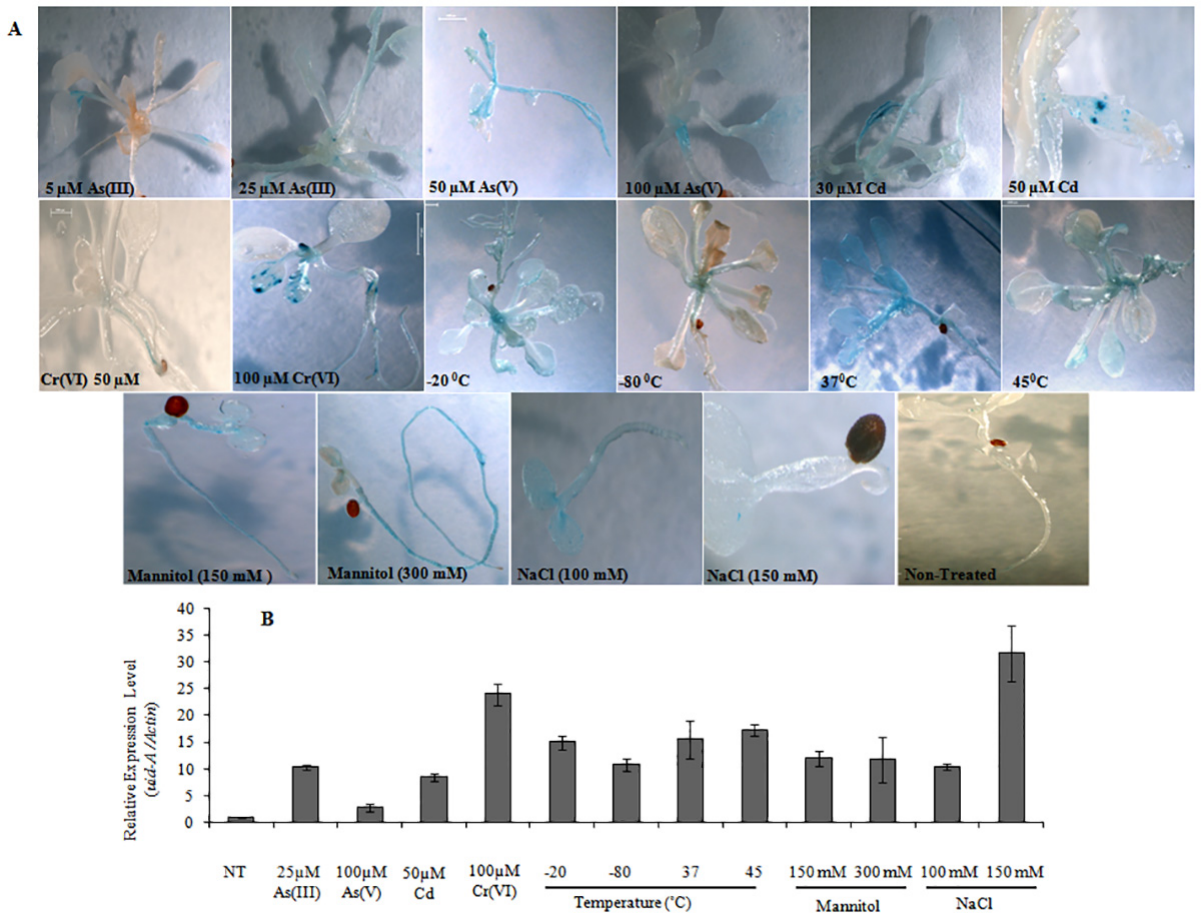
Promoter activity in *Arabidopsis* line expressing ProOs08g01480:*uidA*. (A) Histochemical GUS staining (B) Relative expression level of *uid-A* gene of transgenic seedling (10 d old), grown in ½ MS media supplemented with 5 and 25 μM As(III), 50 and 100 μM As(V), 30 and 50 μM Cd, 50 and 100 μM Cr(VI), 150 and 300 mM mannitol, 100 and 150 mM NaCl. For temperature stress seeds of transgenic line grown in ½ MS media plate and exposed to -20°C, -80°C, 37°C and 45°C for 4 h then allowed to grow in control conditions (10 d). NT represents for non treated transgenic line.

GUS activity was examined in transgenic seedling grown in ½ MS media supplemented with different concentration of metals. Histochemical GUS staining showed significant activity of promoter in *Arabidopsis* seedlings during As(III), As(V), and Cd stress at different concentrations. Interestingly, no significant activity was observed during Cr(VI) exposure in comparison to other HMs stresses at lower concentration (Fig 7a). GUS activity was observed in flowers and roots of *Arabidopsis* plants exposed to different HMs. While in leaf tissue, GUS activity was observed only in the plants exposed to As(III), As(V), Cd stresses. In case of Cr(VI), GUS activity was observed in leaves only in higher concentration. GUS activity was also observed in shoot of *Arabidopsis* during As(III), As(V), Cd and Cr(VI) exposure at higher and lower concentration (Fig L and M in S1 File). Enhanced GUS activity was also found in other abiotic stresses such as temperature, osmotic and salt stress (Fig 7a). Relative expression of *uid-A* gene agreed with the GUS staining patterns (Fig 7b).

## Discussion

Plant CYP enzymes show supremacy in many detoxification pathways and may be amenable for metal detoxification by metabolism [33–34]. In our earlier study, we established that CYP gene family expresses differentially in the rice seedlings during As(V) and As(III) stresses [6]. However, there is limited knowledge about the function of CYP gene family in rice. Therefore to elucidate the function of metal responsive CYP, *Os08g01480* was characterized in this study.

The constitutive expression of *Os08g01480* led to different perceptible changes in growth and morphology such as early bolting and enhanced plant root growth (Fig C in S1 File). Few other studies also portray function of CYPs in regulation plant development [35–36]. Transgenic lines displayed significant increased root length under different HMs and other abiotic stresses in comparison to WT (Figs 1, 2 and 3). Lateral root formation was also promoted in transgenic lines in different abiotic stresses. The promotion of lateral root formation under stress was consistent with the higher expression of the *Os08g01480* gene expression in Line 1 and Line 3. This phenomenon may reflect the requirement of plants for these lateral roots to compensate for the loss of mineral nutrients and water. This phenotype may also represent an adaptive response of plants to abiotic stresses. Transgenic lines showed increased accumulation of total As when grown in As(V) as compared to other HMs. Root length data also showed increased root length in transgenic lines during As(V) stress. In a previous study, higher expression (8.08 fold) of *Os08g01480* was observed in case of As(V) exposure in rice roots [6]. Apart from that, heat map profile shows *Os08g01480* expression was also higher in high As accumulating rice genotypes (Bala, Triguna and BRG-12) [21–22] (Fig B in S1 File). These observation suggested a cross-talk between As accumulation and *Os08g01480* expression during As(V) stress which is further supported by higher expression of phosphate transporter (*AtPHT1*), ABC transporters (*AtABCC1* and *AtABCC2*) in transgenic lines (Fig E in S1 File). It is already known that As(V) enters in plant via phosphate transporter [37]. *AtABCC1* and *AtABCC2* are the long-sought and major vacuolar PC transporters and provide tolerance in *Arabidopsis* [38].

Many researchers reported CYPs play crucial roles in response to heavy metal stresses [13,39,6,7] abiotic stress (salt, cold and osmotic stress) [40] or plant defense response pathways involving salicylic acid and abscisic acid signaling [41]. However, the functions of most of the cytochrome P450 genes are not known. Therefore, it is important to analyze the function of these genes not only to understand the molecular mechanisms of stress- tolerance and response of higher plants but also as a tool for deciphering the crosstalk of genes in response to different physiological processes.

Chlorophyll fluorescence and analyses of PSII is a useful tool for the monitoring of abiotic stresses [42–45]. Different studies predicted that metal, temperature; salt and osmotic stresses alter chlorophyll fluorescence in plants. Mallick and Mohn, [42] demonstrated that metals (Cu, Cr, Ni, Cd and Zn) inhibit PSII photochemistry. Authors showed that these metals affect the maximum quantum yield, photochemical and non-photochemical quenching, and plastoquinone pool. Lu et al. [46] measured physiological changes by photo-inhibition at high salinity. It was concluded that reaction centers of plants were damaged (photochemically inactive), thus PSII had reduced electron transport capacity. In our analysis, both light and dark reactions of photosynthesis were impaired much more in WT than in transgenic *Arabidopsis* plants because performance indices of photochemical Y (NPQ) reactions were much lower in metal stress-exposed plants than the plant grown under control conditions (Fig G in S1 File).

During stress conditions, WT showed lowering of Fv/Fm as compared to transgenic plants, similar findings were observed in tall fescue [47] and pea leaves [48]. This analysis indicates tolerant behavior of *Os08g01480* plants under stress conditions. HM stress slightly affects the maximum photochemical quantum yield of PSII reaction centers. The difference between WT and transgenic lines is clear under As(III) stress. Transgenic lines escape from HM stresses by increasing regulated energy dissipation via xanthophylls cycle. However WT can’t survive due to lowering of Fv/Fm or damage of PSII reaction centers.

Furthermore, to understand the molecular network regulated by *Os08g01480*, pathway studio analysis was carried out (Fig 5). This study indicated that improved root growth and early bolting in the transgenic lines is possibly due to the increased expression of *HY5* and *EDS1*, *COI1*, *YUC4* and *ER* respectively. *HY5* is known positive regulator of ABA signaling [49] and ABA is considered as a key hormone in abiotic stress responses [50]. *YUC4* encodes auxin biosynthesis enzymes and increase in auxin levels and also regulates up-regulation of auxin-responsive genes [51]. From this analysis, it was hypothesized that tolerant response of transgenic lines towards different HMs and abiotic stresses is might be due to the crosstalk of auxin-ABA metabolism which is already known for its role in plant development and defense [52–53]. Auxin regulates plant growth and development which might be intimately linked to plant defense pathway through ABA metabolism [54]. Additionally, increased root length and more lateral roots are also known to be controlled by auxin and ABA, and auxin has direct effect on lateral root initiation [55–57]. Transgenic lines expressing *Os08g01480* showed early germination as well as enhanced growth. This changes might be due to modulated expression of genes involved in hormonal cross-talk which has been observed through pathway analysis. Furthermore expression profile of *COI1* and *BRI1* was higher in L1 in comparison to other transgenic lines may be due to high expression of *Os08g01480* gene in L1 (Fig C in S1 File).

To study transcriptional regulation of gene expression in response to different abiotic stresses promoter analysis was carried out. Several regulatory motifs in promoter region of *Os08g01480* using online tool such as PLACE identified. The identified consensus motifs are known to play role in defense related pathways, pollen development, systematic acquired resistance (SAR), cold response, different stress response, environment response (Table C in S1 File). Promoter activity of *Os08g01480* was also analyzed using histochemical GUS staining. During different developmental stages of rice, promoter activity was predominant during flower development mainly in anther (Fig I in S1 File). Promoter activity of *Os08g01480* was also analyzed under different HM stresses [As(V), As(III), Cd, and Cr(VI)] and other abiotic stresses (cold, heat, salt and osmotic). Significantly high GUS activity was observed in different abiotic stresses in transgenic *Arabidopsis* seedlings (Fig 7). Therefore, from the above results, it could be anticipated that the aggregation of *cis*-acting elements present in the proximal promoter of rice *Os08g01480* predestined and mediated positive transcriptional regulation in growth and development of plant during different abiotic stresses. Additionally, analysis of *Os08g01480* promoter study supports the response of the *Os08g01480* expressing transgenic lines.

## Conclusion

Our study suggests that the expression of *Os08g01480* is differentially modulated during different developmental stages and environmental stresses. *Os08g01480* expression further antecedents the co-regulation of growth and defense related genes. This alteration is the molecular basis for the HM tolerance exhibited by *Os08g01480* expressing transgenic *Arabidopsis*.

## Supporting Information

S1 File Figures A–M, Tables A–C. **Fig A in S1 File. An unrooted, maximum likelihood phylogenetic tree of CYPs**. All CYP sequences of *Arabidopsis* and rice CYP *Os08g01480* was aligned using ClustalW and phylogenetics tree was created using MEGA v5.2. Branch lengths are proportional to evolutionary distance with each other. *Os08g01480* is encircled with red border. **Fig B in S1 File. Expression profile of *Os08g01480***. (A) Expression in roots of two different japonica rice cultivars [Azucena (As(V)-sensitive) and Bala (As(V)-tolerant)] exposed to 13.3 mM concentration of As(V). The detailed description of the datasets utilized for the study is provided in S1 Table. (B) Expression profile in roots of six contrasting rice genotypes exposed to 50 μM concentration of As(V) for 24 h under standard physiological conditions of 16 h light (115 μmol m^-2^ s^-1^) and 8 h dark photoperiod at 25 ± 2°C temperature. HARG and LARG denotes for High arsenic accumulating rice genotypes and Low arsenic accumulating rice genotypes. (C) Differential expression pattern of *Os08g01480* during abiotic Stresses. Expression profile analysis of *Os08g01480* during control conditions (C), cold (CS), drought (DS) and salinity (SS) stresses. The colour scale (representing log signal values) is shown at the top. **Fig C in S1 File. Development of *Os08g01480* expressing *Arabidopsis* lines** (A) Schematic representation of construct used for transformation of *Arabidopsis*. (B) Expression analysis of *Os08g01480* in transgenic lines using semi-quantitative PCR analysis and *actin* is taken as endogenous control. (C) Relative expression analysis of *Os08g01480* in transgenic lines through qRT-PCR analysis. Data are shown as mean ± SD of three biological replicates in each independent experiment. (D) Growth of WT and *Os08g01480* expressing *Arabidopsis* lines (L1, L2 and L3) in soilrite after two-week post germination. **Fig D in S1 File. Growth of WT and *Os08g01480* expressing *Arabidopsis* lines (L1, L2 and L3) in ½ MS plates in control conditions**. (A) Pictorial representation of WT and transgenic lines after 11 d of germination. (B) Root length comparison of WT and transgenic lines after 11 d of germination. All values are the mean of triplicates (±SD) Values marked with similar letters are not significantly (Duncan’s test: p<0.05) different. (C) Pictorial representation of comparative analysis of germination percentage of WT and transgenic lines after 5 d of germination. (D) Percentage germination (recorded after radical emergence) comparison of WT and transgenic lines. All values are the mean of triplicates (±SD) Values marked with similar letters are not significantly (Duncan’s test: p<0.05) different. **Fig E in S1 File. Heavy metal accumulation and relative expression analysis** (A) Heavy metal accumulation was measured in whole seedling as described in materials and method. Seeds of *Arabidopsis thaliana* (WT) and three transgenic lines (L1, L2 and L3) were grown in ½ MS media plates supplemented with 5 μM As(III), 100 μM As(V), 50 μM Cd and 50 μM Cr(VI). All values are the mean of triplicates (±SD) Values marked with similar letters are not significantly (Duncan’s test: p<0.05) different. (B) Expression analysis of *AtPHT1*, *AtABCC1* and *AtABCC2* in *Arabidopsis*. qRT-PCR analysis was carried out in RNA isolated from seedlings of *Arabidopsis thaliana* (WT) and three transgenic lines (L1, L2 and L3) were grown in ½ MS media plates. **Fig F in S1 File. Spider plot of chlorophyll fluorescence in WT and three independent transgenic lines (L1, L2 and L3) in control conditions**. NT represents for no treatment. Spider plot represents relative changes of mean values of selected fluorescence parameters of maximum photosynthetic efficiency (Fv/Fm), photosynthetic yield Y(II), nonphotochemical quenching (NPQ), regulated energy dissipation Y (NPQ), nonregulated heat dissipation Y (NO) and coefficient of photochemical quenching and non photochemical quenching (qN). **Fig G in S1 File. Spider plot of chlorophyll fluorescence** in (A) WT plant (Col-0), (B) Transgenic line (L1), (C) Transgenic line (L2), (D) Transgenic line (L3) during heavy metal stress 100 μM As(III), 200 μM As(V), 200 μM Cd and 400 μM Cr(VI). NT represents for No Treatment. Spider plot represents relative changes of mean values of selected fluorescence parameters of maximum photosynthetic efficiency (Fv/Fm), photosynthetic yield Y(II), nonphotochemical quenching (NPQ), regulated heat dissipation Y (NPQ), and nonregulated heat dissipation Y (NO). Level of significance considered as P≤0.05. **Fig H in S1 File. Spider plot of chlorophyll fluorescence** in (A) WT plant (Col-0), (B) Transgenic line (L1), (C) Transgenic line (L2), (D) Transgenic line (L3) NT represents for No Treatment. Spider plot represents relative changes of mean values of selected fluorescence parameters of maximum photosynthetic efficiency (Fv/Fm), photosynthetic yield Y(II), nonphotochemical quenching (NPQ), regulated energy dissipation Y (NPQ), and nonregulated heat dissipation Y (NO). Level of significance considered as P≤0.05. **Fig I in S1 File. Analysis of expression profile of *Os08g01480***. (A) Expression profile of *Os08g01480* during different developmental stages of rice. (B) Expression profile of *Os08g01480* during different stages of anther development. (C) qRT-PCR analysis of *Os08g01480* during different developmental stages of rice. **Fig J in S1 File. Construct preparation and selection of transgenic lines**. (A) Schematic representation of T-DNA of plant expression construct carrying *Os08g01480* promoter in pBI 121vector, used for *Arabidopsis* transformation. (B) Genomic DNA PCR to confirm presence of 500 bp *Os08g01480* promoter in transgenic lines. **Fig K in S1 File. Promoter activity in *Arabidopsis* line expressing ProOs08g01480:*uidA***. Representative images after GUS staining of (A) seedling (10 d old grown in ½ MS media) (B) Leaf, (C) Shoot, (D) Root, (E) Flowers, and (F) Silique of mature plant show promoter activity grown in pot for 15 d under normal conditions. (G) Relative expression of *uid-A* gene in different tissues of transgenic line grown in pot for 10 d under normal conditions. **Fig L in S1 File. Promoter activity in flower and leaves of *Arabidopsis* line (L1) expressing ProOs08g01480:*uidA* under different heavy metal stresses**. Histochemical GUS staining of (A) flowers and (B) leaves of transgenic mature plants after 15 d of growth in pots under normal conditions supplemented with nutrient media containing different heavy metals As(III) 5 and 25 μM, As(V) 50 and 100 μM, Cd 30 and 50 μM, Cr(VI) 50 and 100 μM. **Fig M in S1 File. Promoter activity in shoots and roots of *Arabidopsis* line (L1) expressing ProOs08g01480:*uidA* under different heavy metal stresses**. Histochemical GUS staining of (A) shoot and (B) roots of transgenic mature plants after 15 d of growth in pots under normal conditions supplemented with nutrient media containing different heavy metals: 5 and 25 As(III) μM, 50 and 100 μM As(V), 30 and 50 μM Cd, 50 and 100 μM Cr(VI). **Table A in S1 File. Details of rice microarray experiments from GEO database used in this study. Table B in S1 File. List of primes. Table C in S1 File. *cis*-regulatory elements present in promoter of *Os08g01480***.
(DOCX)
