# Supplementary material for: Expression of Rice CYP450-Like Gene (Os08g01480) in Arabidopsis Modulates Regulatory Network Leading to Heavy Metal and Other Abiotic Stress Tolerance
Source: PLoS One. 2015 Sep 24;10(9):e0138574. doi: 10.1371/journal.pone.0138574 (PMC4581635; doi:10.1371/journal.pone.0138574)
Supplement: S1 File — Fig A in S1 File. An unrooted, maximum likelihood phylogenetic tree of CYPs. All CYP sequences of Arabidopsis and rice CYP Os08g01480 was aligned using ClustalW and phylogenetics tree was created using MEGA v5.2. Branch lengths are proportional to evolutionary distance with each other. Os08g01480 is encircled with red border. Fig B in S1 File. Expression profile of Os08g01480. (A) Expression in roots of two different japonica rice cultivars [Azucena (As(V)-sensitive) and Bala (As(V)-tolerant)] exposed to 13.3 mM concentration of As(V). The detailed description of the datasets utilized for the study is provided in S1 Table. (B) Expression profile in roots of six contrasting rice genotypes exposed to 50 μM concentration of As(V) for 24 h under standard physiological conditions of 16 h light (115 μmol m-2 s-1) and 8 h dark photoperiod at 25 ± 2°C temperature. HARG and LARG denotes for High arsenic accumulating rice genotypes and Low arsenic accumulating rice genotypes. (C) Differential expression pattern of Os08g01480 during abiotic Stresses. Expression profile analysis of Os08g01480 during control conditions (C), cold (CS), drought (DS) and salinity (SS) stresses. The colour scale (representing log signal values) is shown at the top. Fig C in S1 File. Development of Os08g01480 expressing Arabidopsis lines (A) Schematic representation of construct used for transformation of Arabidopsis. (B) Expression analysis of Os08g01480 in transgenic lines using semi-quantitative PCR analysis and actin is taken as endogenous control. (C) Relative expression analysis of Os08g01480 in transgenic lines through qRT-PCR analysis. Data are shown as mean ± SD of three biological replicates in each independent experiment. (D) Growth of WT and Os08g01480 expressing Arabidopsis lines (L1, L2 and L3) in soilrite after two-week post germination. Fig D in S1 File. Growth of WT and Os08g01480 expressing Arabidopsis lines (L1, L2 and L3) in ½ MS plates in control conditions. (A) Pictorial represe [file pone.0138574.s001.docx]

**Expression of rice CYP450-like gene (*Os08g01480)* in *Arabidopsis* modulates regulatory network leading to heavy metal and other abiotic stress tolerance**

*Arti Rai, Ruchi Singh, Pramod Arvind Shirke, Rudra Deo Tripathi, Prabodh Kumar Trivedi, Debasis Chakrabarty*

Supporting Information S1 File

Figures A–M

Tables A-C

**Figure A. An unrooted, maximum likelihood phylogenetic tree of CYPs.** All CYP sequences of Arabidopsis and rice CYP Os08g01480 was aligned using ClustalW and phylogenetics tree was created using MEGA v5.2. Branch lengths are proportional to evolutionary distance with each other. Os08g01480 is encircled with red border.

**Figure B** Expression profile of *Os08g01480* (A) Expression in roots of two different japonica rice cultivars [Azucena (As(V)-sensitive) and Bala (As(V)-tolerant)] exposed to 13.3 mM concentration of As(V). The detailed description of the datasets utilized for the study is provided in Supplementary Table S1. (B) Expression profile in roots of six contrasting rice genotypes exposed to 50 µM concentration of As(V) for 24 h under standard physiological conditions of 16 h light (115 μmol m^-2^ s^-1^) and 8 h dark photoperiod at 25 ± 2°C temperature. HARG and LARG denotes for High arsenic accumulating rice genotypes and Low arsenic accumulating rice genotypes. (C) Differential expression pattern of *Os08g01480* during abiotic Stresses. Expression profile analysis of *Os08g01480* during control conditions (C), cold (CS), drought (DS) and salinity (SS) stresses. The colour scale (representing log signal values) is shown at the top.

**Figure C. Development of *Os08g01480* expressing *Arabidopsis* lines** (A) Schematic representation of construct used for transformation of *Arabidopsis.* (B) Expression analysis of *Os08g01480* in transgenic lines using semi-quantitative PCR analysis and *actin* is taken as endogenous control. (C) Relative expression analysis of *Os08g01480* in transgenic lines through qRT-PCR analysis. Data are shown as mean ± SD of three biological replicates in each independent experiment. (D) Growth of WT and *Os08g01480* expressing *Arabidopsis* lines (L1, L2 and L3) in soilrite after two-week post germination.

**Figure D. Growth of WT and Os08g01480 expressing Arabidopsis lines (L1, L2 and L3) in ½ MS plates in control conditions.** (A) Pictorial representation of WT and transgenic lines after 11 d of germination. (B) Root length comparison of WT and transgenic lines after 11 d of germination. All values are the mean of triplicates (±SD).Values marked with similar letters are not significantly (Duncan’s test: p<0.05) different. (C) Pictorial representation of comparative analysis of germination percentage of WT and transgenic lines after 5 d of germination. (D) Percentage germination (recorded after radical emergence) comparison of WT and transgenic lines. All values are the mean of triplicates (±SD).Values marked with similar letters are not significantly (Duncan’s test: p<0.05) different.

**Figure E. Heavy metal accumulation and relative expression analysis** (A) Heavy metal accumulation was measured in whole seedling as described in materials and method. Seeds of *Arabidopsis thaliana* (WT) and three transgenic lines (L1, L2 and L3) were grown on ½ MS media plates supplemented with 5 µM As(III), 100 µM As(V), 50 µM Cd and 50 µM Cr(VI). All values are the mean of triplicates (±SD).Values marked with similar letters are not significantly (Duncan’s test: p<0.05) different. (B) Expression analysis of *AtPHT1*, *AtABCC1* and *AtABCC2* in *Arabidopsis*. qRT-PCR analysis was carried out in RNA isolated from seedlings of *Arabidopsis thaliana* (WT) and three transgenic lines (L1, L2 and L3) were grown on ½ MS media plates.

**Figure F. Spider plot of chlorophyll fluorescence in WT and three independent transgenic lines (L1, L2 and L3) in control conditions.** NT represents for no treatment. Spider plot represents relative changes of mean values of selected fluorescence parameters of maximum photosynthetic efficiency (Fv/Fm), photosynthetic yield Y(II), nonphotochemical quenching (NPQ), regulated energy dissipation Y (NPQ), nonregulated heat dissipation Y (NO) and coefficient of photochemical quenching and non photochemical quenching (qN).

**Figure G. Spider plot of chlorophyll fluorescence** in (A) WT plant (Col-0), (B) Transgenic line (L1), (C) Transgenic line (L2), (D) Transgenic line (L3) during heavy metal stress 100 µM As(III), 200 µM As(V), 200 µM Cd and 400 µM Cr(VI). NT represents for No Treatment. Spider plot represents relative changes of mean values of selected fluorescence parameters of maximum photosynthetic efficiency (Fv/Fm), photosynthetic yield Y(II), nonphotochemical quenching (NPQ), regulated heat dissipation Y (NPQ), and nonregulated heat dissipation Y (NO). Level of significance considered as P≤0.05.

**Figure H. Spider plot of chlorophyll fluorescence** in (A) WT plant (Col-0), (B) Transgenic line (L1), (C) Transgenic line (L2), (D) Transgenic line (L3) NT represents for No Treatment. Spider plot represents relative changes of mean values of selected fluorescence parameters of maximum photosynthetic efficiency (Fv/Fm), photosynthetic yield Y(II), nonphotochemical quenching (NPQ), regulated energy dissipation Y (NPQ), and nonregulated heat dissipation Y (NO). Level of significance considered as P≤0.05.

**Figure I. Analysis of expression profile of *Os08g01480*.** (A) Expression profile of *Os08g01480* during different developmental stages of rice. (B) Expression profile of *Os08g01480* during different stages of anther development. (C) qRT-PCR analysis of *Os08g01480* during different developmental stages of rice.

**Figure J.** **Construct preparation and selection of transgenic lines.** (A) Schematic representation of T-DNA of plant expression construct carrying *Os08g01480* promoter in pBI 121vector, used for *Arabidopsis* transformation. (B) Genomic DNA PCR to confirm presence of 500 bp *Os08g01480* promoter in transgenic lines.

**Figure K.** **Promoter activity in *Arabidopsis* line expressing ProOs08g01480*:uidA*.** Representative images after GUS staining of (A) seedling (10 d old grown in ½ MS media) (B) Leaf, (C) Shoot, (D) Root, (E) Flowers, and (F) Silique of mature plant show promoter activity grown in pot for 15 d under normal conditions. (G) Relative expression of *uid-A* gene in different tissues of transgenic line grown in pot for 10 d under normal conditions.

**Figure L.**  **Promoter activity in flower and leaves of *Arabidopsis* line (L1) expressing ProOs08g01480*:uidA* under different heavy metal stresses.** Histochemical GUS staining of (A) flowers and (B) leaves of transgenic mature plants after 15 d of growth in pots under normal conditions supplemented with nutrient media containing different heavy metals As(III) 5 and 25 µM, As(V) 50 and 100 µM, Cd 30 and 50 µM, Cr(VI) 50 and 100 µM.

**Figure M. Promoter activity in shoots and roots of *Arabidopsis* line (L1) expressing ProOs08g01480*:uidA* under different heavy metal stresses.** Histochemical GUS staining of (A) shoot and (B) roots of transgenic mature plants after 15 d of growth in pots under normal conditions supplemented with nutrient media containing different heavy metals: 5 and 25 As(III) µM, 50 and 100 µM As(V), 30 and 50 µM Cd, 50 and 100 µM Cr(VI).

| **Table A.** **Details of rice microarray experiments from GEO database used in this study**. | | | |  |
| --- | --- | --- | --- | --- |
| **S. No.** | **Series accession No.** | **Sample** | **Description** |  |
| 1. | GSE4471 | Rice Azucena 0ppm As(V) (Control)  Rice Azucena 1ppm As(V)  Rice Bala 0ppm As(V) (Control)  Rice Bala 1ppm As(V) | Gene expression data from rice roots (variety Azucena and Bala) grown in hydroponics for one week with 1ppm and 0ppm As(V). | |
| 2. | GSE6893 | Root, 7-day-old Seedling  Mature Leaf (ML)  Young Leaf (YL)  SAM (Shoot apical meristem)  Young inflorescence (P1, upto 3 cm)  Inflorescence (P2, 3 - 5 cm)  Inflorescence (P3, 5 - 10 cm)  Inflorescence (P4, 10 - 15 cm)  Inflorescence (P5, 15 - 22 cm)  Inflorescence (P6, 22 - 30 cm)  Seed (S1, 0 - 2 dap)  Seed (S2, 3 - 4 dap)  Seed (S3, 5 - 10 dap)  Seed (S4, 11 - 20 dap)  Seed (S5, 21 - 29 dap)  (dap represents day after pollination) | The spatial and temporal gene expression in various tissues/organs and developmental stages of rice using microarray technology is used to identify the genes differentially expressed during various stages of reproductive development. | |
| 3. | GSE7951 | Stigma  Ovary | Rice stigma-specific gene expression profiles through comparing genome-wide expression patterns of hand dissected unpollinated stigma at anthesis with seven tissues including seedling shoot, seedling root, mature anther, ovary at anthesis, seeds of five days after pollination, 10-day-old embryo, 10-day-old endosperm as well as suspension cultured cells studied by using 57K Affymetrix rice whole genome array. | |
| 4. | GSE14304 | Anther, hypodermal archesporial cells forming stage (An1)  Anther, Pollen mother cells at pre-meiotic s/g2 stage (Mei1)  Anther, Pollen mother cells at meiotic leptotene stage (M1)  Anther, Pollen mother cells at meiotic zygotene-pachytene stage (M2)  Anther, Pollen mother cells at meiotic diplotene-tetrad stage (M3)  Anther, Uni-nucleated gametopyte stage (P1)  Anther, bi-cellular gametopyte stage (P2)  Anther, tri-cellular mature pollen stage (P3) | Gene expression data from developing anther. | |
| 5. | GSE6901 | Rice Seedlings | Seven-day-old light-grown rice seedlings grown under controlled conditions and those subjected to various abiotic stress conditions were used for RNA extraction and hybridization on Affymetrix microarrays. Three biological replicates of each sample were used for microarray analysis. For salt treatment (SS), the rice seedlings were transferred to a beaker containing 200 mM NaCl solution for 3 h. For desiccation (DS), rice seedlings were dried for 3 h between folds of tissue paper at 28±1 degree C, in a culture room. For cold treatment (CS), the seedlings were kept at 4±1 degree C for 3 h. The seedlings kept in water for 3 h, at 28±1 degree C, served as control (Seedling). | |

| **Table B. List of primes** | |
| --- | --- |
| *(GBF3) AT2G46270*F | AAGTCGA GAGGGAACTC C AAC |
| *(GBF3) AT2G46270*R | AGAGAGTATAGCTCCAGATCC |
| *(EDS1) AT3G48090*F: | ATGT TTACCTTGAG CCTCGTTG |
| *(EDS1) AT3G48090*R: | TCCGAGGGACAATATCGAATC |
| *(HY5) AT5G11260*F: | AGCTCAGCAAGCAAGAGAGAG |
| *(HY5) AT5G11260*R: | AGCATCTGGTTCTCGTTCTG |
| *(ER) AT2G26330*F: | GCCA A CTGTCTCAGTCTCACTG |
| *(ER) AT2G26330*R: | AGCCAACTACCGCAAAGACCAG |
| *(COI1) AT2G39940*F | GAAACTGAAGACCATTGTACGC |
| *(COI1) AT2G39940*R | CTTGTTCATCTGCACCGCGTTC |
| *(PAD4) AT3G52430*F: | TCCATCCACGACCTCGTTCC |
| *(PAD4) AT3G52430*R: | TGAGTTGCTGTGGTGTTGAGG |
| *(AOS) AT5G42650*F: | AAGTCAAAGCCGGTGAAATG |
| *(AOS) AT5G42650*R: | ACAACACATGCCTCAAAAGC |
| *(YUC4) AT5G11320*F: | TGTAGATACAATGCTCTGCCTC |
| *(YUC4) AT5G11320*R: | CCATTTCAGTAGTGTCATGGC |
| *(CYP707A1) AT4G19230*F: | GGGAGGGAACAATGATCAACAC |
| *(CYP707A1) AT4G19230*R: | CTCGAGAATGTAGTAGCATCG |
| *(BRI1) AT4G39400*F: | AGTTACCGATGGATACGTTGTTG |
| *(BRI1) AT4G39400*R: | GAGATCTAACGTTAGCAACGAAGC |
| *(CYP82C2) AT4G31970*F: | CATGGGTTACGATTGTGCTG |
| *(CYP82C2) AT4G31970*R: | CACGGACATGCTTGAGCATCTG |
| *(CYP72C1) AT1G17060*F: | TGG ACTCACTGTCATGACTTAAC |
| *(CYP72C1) AT1G17060*R: | TTGAGCCAGGAATGTAGACG |
| *AtPHT1*F | [CCTCAACTCTCCAGAGAAGTTC](http://cgi-www.daimi.au.dk/cgi-chili/primique/showprimers.py?l1=22&l2=21&g=camel28&p1=19&p2=48&t=/tmp/tmpbqhGif.dir.primique&h=gi%7C145358803%7Cref%7CNM_123701.3%7C%20Arabidopsis%20thaliana%20inorganic%20phosphate%20transporter%201-1%20mRNA%2C%20complete%20cds&pagemax=25&minind=0&pmin=50&maxrepeat=2&gcmaxtail=60&tmin=50.0&mintid=Wed%20Jul%20%209%2011:41:11%202014&min=18&max=22&tmax=60.0&gcmin=40&maxspecificity=on&minID=114054&maxautohybrid=on&maxcrosshybrid=on&maxsugg=2&gcterminal=on&pmax=150&gcmax=60&gcmintail=40&maxtmdiff=2.0) |
| *AtPHT1*R | [TCTTAATCAGCTTGGCAGGAG](http://cgi-www.daimi.au.dk/cgi-chili/primique/showprimers.py?l1=22&l2=21&g=camel28&p1=19&p2=48&t=/tmp/tmpbqhGif.dir.primique&h=gi%7C145358803%7Cref%7CNM_123701.3%7C%20Arabidopsis%20thaliana%20inorganic%20phosphate%20transporter%201-1%20mRNA%2C%20complete%20cds&pagemax=25&minind=0&pmin=50&maxrepeat=2&gcmaxtail=60&tmin=50.0&mintid=Wed%20Jul%20%209%2011:41:11%202014&min=18&max=22&tmax=60.0&gcmin=40&maxspecificity=on&minID=114054&maxautohybrid=on&maxcrosshybrid=on&maxsugg=2&gcterminal=on&pmax=150&gcmax=60&gcmintail=40&maxtmdiff=2.0) |
| *AtABCC1*F | CCGCAGAAATCCTCTTGGTCTTGATG |
| *AtABCC1*R | GTGAATCATCACCGTTAGCTTCTCTGG |
| *AtABCC2*F | AGCGTGCCAAAGATGACTCACACCAC |
| *AtABCC2*R | TACTTATCACGAAGAACACAACAGGG |
| *Os08G01480*RTF | AGCCAACAATGGCATCCTAC |
| *Os08G01480*RTR | GCGGAGAAGCATTAGGTCAG |
| *Os08G01480*F1 | TGACAGGTCTCACACACCAACCTC |
| *Os08G01480*R1 | CCTGCGAGGTGTAGCCGAAGCATC |
| *Os08G01480*F2 | AGACACTAGAGAGATTAGGCAG |
| *Os08G01480*R2 | GAAGCATCTCCTTTATCACTGC |
| M13F | GTAAAACGACGGCCAGT |
| M13R | CAGGAAACAGCTATGAC |
| *Os08g01480Xba*I | AGACAGTCTAGAGATTAGGCAGCCATGGCTG |
| *Os08g01480Sac*I | GAAGGAGCTCCTTTATCACTGCCTTTA |
| Pro*Os08g01480*F1 | GGAAAATGGAGGAGGTGACA |
| Pro*Os08g01480*R1 | GGTGCTGGTGCAAGAGTAGTAG |
| Pro*Os08g01480*F2 | GAGAAAGTGACATGACAAGAGGTG |
| Pro*Os08g01480*R2 | GGCACTAGCATCACAGCCATGGCTG |
| Pro*Os08g01480 BamH*I | ATTGGATCCTAGCATCACAGCCATGGCTGCC |
| *ProOs08G01480 HindIII* | ATAGACGAAGCTTAAAGAGGTCCGT |
| *OsActinF* | GATGGATCCTCCAATCCAGACACTGTA |
| *OsActinF* | GTATTGTGTTGGACTCTGGTGATGGTGT |
| *OsUbiqutinF* | GACGGACGCACCCTGGCGAACTAC |
| *OsUbiqutinF* | TGCTGCCAATTACCATATACCACGAC |
| *AtActinF* | ATGACATGGAGAAGATCTGGCATCA |
| *AtActinR* | AGCCTGGATGGCAACATACATAGC |

| **Table C. *cis*-regulatory elements present in promoter of *Os08g01480*** | | |
| --- | --- | --- |
| PLACE_ID | MOTIF | DESCRIPTION |
| DOFCOREZM | AAAG | Dof1 and Dof2 transcription factors are associated with expression of multiple genes involved in carbon metabolism in maize |
| GTGANTG10 | GTGA | "GTGA motif" found in the promoter of the tobacco late pollen gene g10 (pollen development) |
| WRKY71OS | TGAC | "A core of TGAC-containing W-box" of, e.g., Amy32b promoter Binding site of rice WRKY71, a transcriptional repressor of the gibberellin signaling (pathway; Parsley WRKY proteins bind specifically to TGAC-containing W box elements within the Pathogenesis-Related Class10 (PR-10) genes (Defence related pathway) |
| POLLEN1LELAT52 | AGAAA | One of two co-dependent regulatory elements responsible for pollen specific activation of tomato (Pollen Development) |
| TATABOX5 | TTATTT | "TATA box"; TATA box found in the 5'upstream region of pea glutamine synthetase gene |
| WBOXATNPR1 | TTGAC | "W-box" found in promoter of *Arabidopsis thaliana* NPR1 gene; Located between +70 and +79 in tandem; They were recognized specifically by salicylic acid (SA)-induced WRKY DNA bindingproteins (Defence,SAR) |
| CBFHV | RYCGAC | Binding site of barley CBF1, and also of barley CBF2; CBF DE = C-repeat (CRT) binding factors; CBFs are also known as DE dehydration-responsive element (DRE) binding proteins (DREBs) (Cold Response) |
| PREATPRODH | ACTCAT | "PRE (Pro- or hypoosmolarity-responsive element) found in the promoter region of proline dehydrogenase (ProDH) gene in *Arabidopsis* (stress response) |
| MYCCONSENSUSAT | CANNTG | MYC recognition site found in the promoters of the dehydration-responsive gene rd22 and many other genes in *Arabidopsis*; Binding site of ATMYC2 (stress response) |
| BOXLCOREDCPAL | ACCWWCC | Consensus of the putative "core" sequences of box-L-like sequences in carrot PAL1 promoter region; DCMYB1 bound to these sequences in vitro (Environmental Stress) |
| CCAATBOX1 | CCAAT | CONSTANS and the CCAAT Box Binding Complex Share a Functionally Important Domain and Interact to Regulate Flowering of *Arabidopsis* (Flower development) |
| MYB2CONSENSUSAT | YAACKG | MYB recognition site found in the promoters of the dehydration-responsive gene rd22 and many other genes in *Arabidopsis* (ABA signaling in stress-response and seed development). |
